# CT-Based Radiomics to Differentiate Pelvic Rhabdomyosarcoma From Yolk Sac Tumors in Children

**DOI:** 10.3389/fonc.2020.584272

**Published:** 2020-11-24

**Authors:** Xin Chen, Yan Huang, Ling He, Ting Zhang, Li Zhang, Hao Ding

**Affiliations:** Department of Radiology, Chongqing Key Laboratory of Pediatrics, Ministry of Education Key Laboratory of Child Development and Disorders, National Clinical Research Center for Child Health and Disorders (Chongqing), China International Science and Technology Cooperation Base of Child Development and Critical Disorders, Children’s Hospital of Chongqing Medical University, Chongqing, China

**Keywords:** rhabdomyosarcoma, yolk sac tumor, CT, radiomics, differential diagnosis

## Abstract

**Background:**

The purpose of this study was to investigate the role of CT radiomics features combined with a support vector machine (SVM) model in potentially differentiating pelvic rhabdomyosarcoma (RMS) from yolk sac tumors (YSTs) in children.

**Methods:**

A total of 94 patients with RMS (n = 49) and YSTs (n = 45) were enrolled. Non-enhanced phase (NP), arterial phase (AP), and venous phase (VP) images were retrieved for analysis. The volumes of interest (VOIs) were constructed by segmenting tumor regions on CT images to extract radiomics features. Datasets were randomly divided into two sets including a training set and a test set. In the training set, the least absolute shrinkage and selection operator (LASSO) algorithm was used to screen out the optimal radiomics features that could distinguish RMS from YSTs, and the features were combined with the SVM algorithm to build the classifier model. In the testing set, the areas under the receiver operating characteristic (ROC) curves (AUCs), accuracy, specificity, and sensitivity of the model were calculated to evaluate its diagnostic performance. The clinical factors (including age, sex, tumor site, tumor volume, AFP level) were collected.

**Results:**

In total, 1,321 features were extracted from the NP, AP, and VP images. The LASSO regression algorithm was used to screen out 23, 26, and 17 related features, respectively. Subsequently, to prevent model overfitting, the 10 features with optimal correlation coefficients were retained. The SVM classifier achieved good diagnostic performance. The AUCs of the NP, AP, and VP radiomics models were 0.937 (95% CI: 0.862, 0.978), 0.973 (95% CI: 0.913, 0.996), and 0.855 (95% CI: 0.762, 0.922) in the training set, respectively, which were confirmed in the test set by AUCs of 0.700 (95% CI: 0.328, 0.940), 0.800 (95% CI: 0.422, 0.979), and 0.750 (95% CI: 0.373, 0.962), respectively. The difference in sex, tumor volume, and AFP level were statistically significant (P < 0.05).

**Conclusions:**

The CT-based radiomics model can be used to effectively distinguish RMS and YST, and combined with clinical features, which can improve diagnostic accuracy and increase the confidence of radiologists in the diagnosis of pelvic solid tumors in children.

## Background

Rhabdomyosarcoma (RMS) is a malignant tumor originating from primitive mesenchymal cells that have the potential to differentiate into striated muscle cells. RMS is the most common soft tissue sarcoma in childhood (1), and it is also the third most common extracranial solid tumor in children, behind neuroblastoma and nephroblastoma (2). The clinical manifestations and laboratory tests of the disease lack of specificity, and there are few radiology reports about RMS (3, 4). Furthermore, RMS has the general radiological appearance of soft tissue tumors, making it difficult to distinguish from other soft tissue malignancies (4). Therefore, it is difficult for radiologists to diagnose RMS correctly before surgery. During the course of routine radiology diagnosis, 54% (20/37) of RMS cases in this study were misdiagnosed as yolk sac tumor (YST) or were difficult to distinguish from YST. RMS in the pelvis of children can be misdiagnosed as YST, which is the most common tumor among pelvic germ cell tumors (5). If a pelvic mass is present, RMS should be considered only when a laboratory examination of alpha fetoprotein (AFP) is used to exclude the mass as a germ cell tumor. At present, biopsy is the only way to confirm the diagnosis of RMS, but only a small part of the tissue can be sampled, and biopsy is invasive, which may lead to complications for some patients. Although RMS and YST are both malignant tumors, RMS has high rates of recurrence and malignant transformation and a poor prognosis (6). There are some differences in the treatment of the two tumors. YSTs are more sensitive to preoperative neoadjuvant chemotherapy than RMS, which are mainly treated by neoadjuvant chemotherapy combined with the surgery. RMS are mainly treated by surgery combined with postoperative adjuvant chemotherapy. Therefore, studies on new radiological methods to effectively identify the two tumors are essential for accurate treatment and assessments of patient prognosis.

In 2012, the Dutch scholars Lambin et al. (7) first proposed “radiomics,” which can convert traditional radiological images into data that can be mined. The extracted high-throughput radiomics features can quantify the spatial-temporal heterogeneity of tumor tissue and provide more objective information beyond visual evaluations. In addition, machine learning has been introduced for further statistical analysis to achieve a more accurate diagnosis or prediction model. In recent years, as a non-invasive and reproducible radiological analysis method, radiomics has been extensively applied for adult diseases (8–14). Compared with the radiomics study of adult diseases, reports of radiomics in children’s diseases are rare. MRI-based radiomics has value in the diagnosis and differential diagnosis of posterior fossa tumors in children (15–17). With regard to radiomics of children’s abdominal tumors, at present, there is only one study (18), which found that histogram parameters (90th percentile of D *, mean value of f, etc.) based on MRI with intravoxel incoherent motion diffusion-weighted imaging (IVIM-DWI) can help to distinguish retroperitoneal neuroblastoma from nephroblastoma in children. This study is the first to apply CT-based radiomics to differentiate pelvic RMS tumors from YSTs in children. To the best of our knowledge, this has not been reported in any published radiology study.

## Methods

### Patients

This study was approved by the Ethics Committee of the Children’s Hospital Affiliated with Chongqing Medical University, and the requirement for written informed consent was waived. The medical record management system and radiology picture archiving and communication system (PACS) of our department were searched from January 2013 to March 2020, and 94 patients with RMS (n = 49) and YST (n = 45) were recruited according to the inclusion and exclusion criteria. The inclusion criteria were as follows: 1) patients underwent abdominal contrast-enhanced CT less than 2 weeks before surgery, and the CT images were clear and usable; 2) pediatric patients with pathologically proven pelvic RMS or YST; and 3) on CT, the tumors appeared as a solid mass. The exclusion criteria were as follows: 1) patients with mixed YST, which contained teratoma (calcifications and adipose tissue) components (5) and 2) patients with bladder RMS (characteristic CT manifestations).

### CT Examination

All RMS and YST patients underwent abdominal three-phase CT scans, and non-enhanced phase (NP), arterial phase (AP), and venous phase (VP) images were acquired. CT examination was performed using a LightSpeed VCT 64-slice CT (GE Healthcare, USA) scanner. The scan extended down to the level of the lower margin of the pubic symphysis. The CT acquisition parameters were as follows: tube voltage of 120 kV, tube current of 200 mAs, pitch of 0.984:1, slice thickness of 5.0 mm, and slice interval of 5.0 mm. After conventional non-enhanced scanning, the contrast agent iohexol (350 µg/ml, 1.5 ml/kg) was injected into the elbow vein through a high-pressure syringe at a flow rate of 1–3 ml/s. The AP and VP images were obtained at 25–30 and 65–70 s, respectively.

### Tumor Segmentation and Image Preprocessing

**Figure 1:**
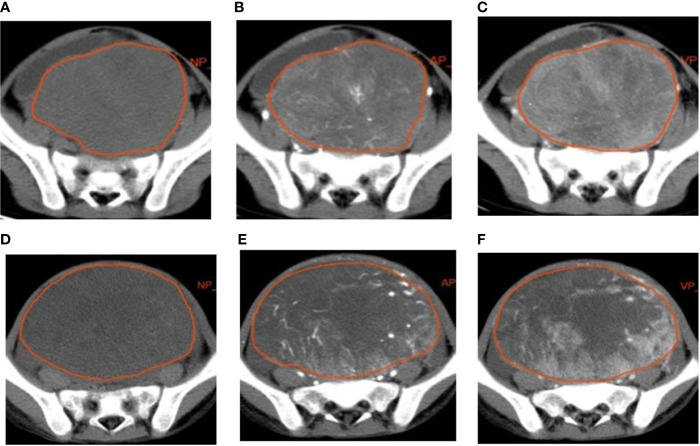
Examples of manual segmenting and contouring of regions of interests (ROIs) of rhabdomyosarcoma (RMS) and yolk sac tumor (YST). Outline of the ROI on one slice of an RMS on non-enhanced phase (NP) **(A)**, arterial phase (AP) **(B)**, and venous phase (VP) **(C)** images; outline of the ROI on one slice of a YST on NP **(D)**, AP **(E)**, and VP **(F)** images.

The lesions were manually delineated on all slices using a radiomics analysis platform [Radcloud, Huiying Medical Technology (Beijing, China) Co., Ltd.] (**Figure 1**). Two radiologists (with over 5 and 10 years of diagnostic experience) delineated and reviewed the regions of interest (ROIs) of the NP, AP, and VP images, and the computer fused the two-dimensional ROIs of the tumor to obtain the three-dimensional volume of interest (3D VOI) of the tumor.

To minimize CT intensity changes and obtain more stable radiomics features, we normalized the intensity of the image using the following formula (where *x* represents the original intensity; *f*(*x*) represents the normalized intensity; μ indicates the average value; σ refers to variance; and *s* is an optional scaling ratio, which has been set to 1 by default).

$$f(x)=\frac{s(x-\mu_{x})}{\sigma_{x}}$$

### Feature Extraction and Standardization

We used Python software (PyRadiomics, v2.2.0) for feature extraction. A total of 1,321 quantitative radiomics features were extracted from the VOIs based on NP, AP, and VP images and could be classified into two categories as follows: 1) first-order statistics, such as peak value, mean value, and variance, which were used to quantitatively describe the distribution of voxel intensity on CT images and 2) texture features, such as gray level cooccurrence matrix (GLCM), gray level run length matrix (GLRLM), and gray level size zone matrix (GLSZM), which were used to quantify the heterogeneity of the selected area. In addition, a variety of filters, such as the logarithm, exponential, gradient, square, square root, local binary patterns (LBPs), and wavelet (including wavelet-LHL, wavelet-LHH, wavelet-HLL, wavelet-LLH, wavelet-HLH, wavelet-HHH, wavelet-HHL, and wavelet-LLL), were used to calculate the first-order statistics and texture features of the transformed images (11). Moreover, before feature selection, the feature values were standardized to [0, 1] to avoid features with a large value interval dominating features with a small value interval (19).

### Feature Selection and Model Construction

The study cohort was randomly divided into two subsets, a training set and testing set, in a proportion of 9:1. First, to reduce the model redundancy, the least absolute shrinkage and selection operator (LASSO) method was used to extract the effective feature values that were closely related to the difference between RMS and YSTs in the training set. Then, to avoid overfitting the model, the 10 most valuable features were screened out by the size of the feature correlation coefficient. Finally, a support vector machine (SVM) model was established based on the extracted optimal features, and the prediction model was verified in the testing set.

### Statistical Analysis

Statistical analysis was performed using Python software (PyRadiomics, v2.2.0) and SPSS (Version 22.0, IBM). Using the pathological results as the gold standard, we calculated the sensitivity and specificity of the SVM model, plotted the receiver operating characteristic (ROC) curve, and calculated the area under the ROC curve (AUC), thus evaluating the prediction performance of the model.

The chi-square test or Fisher’s exact test were used to compare the differences in count data between the two groups, and the independent samples t-test or a non-parametric test were used to compare the differences in measurement data. A value of P < 0.05 was considered statistically significant.

## Results

### Patient Characteristics

A total of 94 patients were enrolled in this study. The 49 patients in the RMS group included 21 males and 28 females, aged 0.5 to 11.7 years, with an average age of 3.9 ± 2.6 years. The tumor sites of the RMS patients included 7 cases of tumors in the perianal area or sacral tail, 4 cases of tumors in the vagina, and 38 cases of tumors in the pelvic (abdominal) cavity. There were 45 patients in the YST group, including 3 males and 42 females, aged 0.4 to 12 years, with an average age of 5.4 ± 4.3 years. The tumor sites of YST patients included 12 cases of tumors in the perianal area or sacral tail, 5 cases of tumors in the vagina, and 28 cases of tumors in the pelvic (abdominal) cavity. The basic clinical data and comparison results of the two groups of patients are shown in **Table 1**. The differences in age and tumor location between the two groups were not statistically significant (P > 0.05), but the difference in sex, tumor volume, and AFP level were statistically significant (P < 0.05). The patient characteristics of the training and testing sets are shown in **Table 2**, and there were no significant differences between the two cohorts.

**Table 1:** Clinical data of patients with rhabdomyosarcoma (RMS) and yolk sac tumors (YST).

Patient characteristics	RMS group	YST group	P value
n	49	45	
Age (x ± s, median, years)	3.9 ± 2.6, 2.5	5.4 ± 4. 3, 4	0.051
Gender			0.001
Male	21	4	
Female	28	42	
Tumor site			0.243
Perianal area or sacral tail	7	12	
Vagina	4	5	
Pelvic (abdominal) cavity	38	28	
Tumor volume (mm^3^)	211,019.24	370,145.75	0.001
*AFP (x ± s)	0.25 ± 0.27	4.076 ± 0.91	<0.001

RMS, rhabdomyosarcoma; YST, yolk sac tumor). *The data of AFP were log10 transformed to ensure the normality.

**Table 2:** Patient characteristics in the training and testing sets.

Patient characteristics	Training cohort	Testing cohort	*P* value
Age (median)	6	6	0.462
Gender			0.577
Male	21	3	
Female	64	6	
Pathology			0.831
RMS group	44	5	
YST group	41	4	

### Feature Extraction and Selection

**Figure 2:**
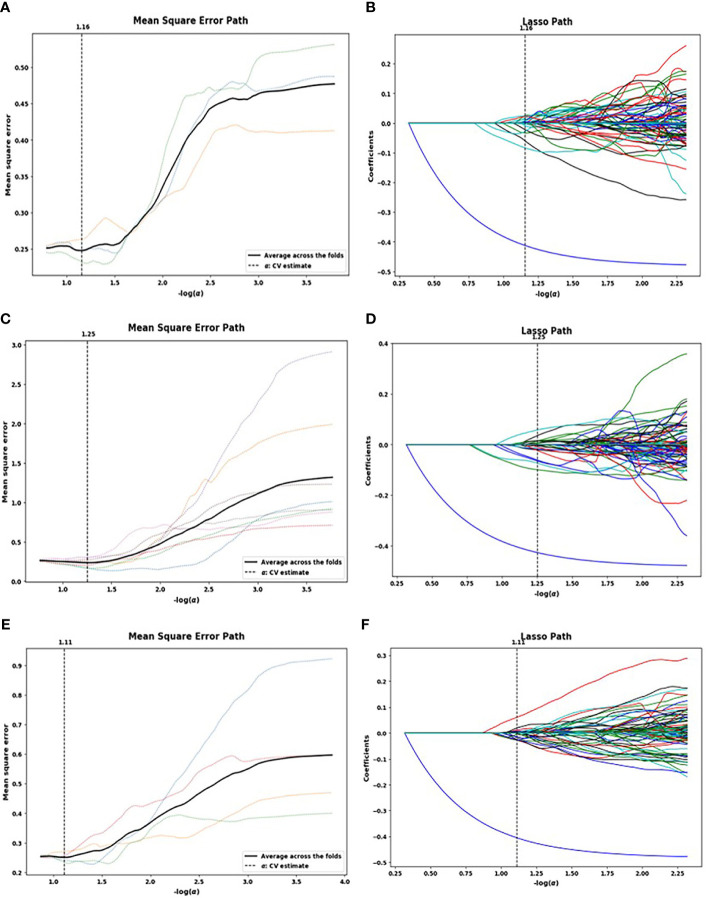
Lasso algorithm for feature selection on non-enhanced phase (NP) **(A, B)**, arterial phase (AP) **(C, D)**, and venous phase (VP) **(E, F)** images. The optimal *a* parameters of the least absolute shrinkage and selection operator (LASSO) model were determined [NP: -log(*a*) = 1.16; AP: -log(*a*) = 1.25; VP: -log(*a*) = 1.11]. Features that correspond to the optimal *a* value were extracted.

A total of 1,321 radiomics features were extracted from each patient’s NP, AP, and VP images. The LASSO algorithm was used to reduce the dimensionality of the above high-dimensional features based on the optimal parameters (**Figure 2**), and 23, 26, and 17 related features were screened from the images, respectively. However, to prevent SVM model overfitting, the 10 features with the highest correlation coefficients were retained (**Table 3**). These features were statistical features of intensity and texture features, and four statistical features of intensity and six texture features were selected from the NP images, including first order, GLSZM, GLDM, and GLRLM. Four statistical features of intensity and six texture features were selected from the AP images, including first order, GLSZM, GLCM, and GLRLM, and six statistical features of intensity and four texture features were selected from the VP images, including first order, GLSZM and GLRLM.

**Table 3:** The 10 optimal features selected by the least absolute shrinkage and selection operator (LASSO) algorithm for each CT phase.

	NP	AP	VP
1	wavelet-HHH_gldm_ HighGrayLevelEmphasis	wavelet-HLH_glszm_ SizeZoneNonUniformityNormalized	wavelet-LHL_glszm_ ZoneEntropy
2	wavelet-HLL_ firstorder_Maximum	wavelet-HHL_glszm_ SizeZoneNonUniformityNormalized	wavelet-LHL_glrlm_ GrayLevelVariance
3	square_firstorder_Skewness	wavelet-HHL_glcm_ Autocorrelation	wavelet-LHH_firstorder_ Median
4	wavelet-LLH_gldm_ DependenceEntropy	wavelet-LLH_ firstorder_Skewness	wavelet-HLL_firstorder_ Maximum
5	square_firstorder_MeanAbsoluteDeviation	wavelet-HHH_glrlm_ LowGrayLevelRunEmphasis	wavelet-HLH_glszm_ HighGrayLevelZoneEmphasis
6	wavelet-HLH_ glszm_ZoneEntropy	wavelet-LHL_ firstorder_Energy	wavelet-HHL_firstorder_ Skewness
7	wavelet-LHL_glszm_ SmallAreaHighGrayLevelEmphasis	wavelet-HLH_ glcm_JointEnergy	gradient_firstorder_Skewness
8	wavelet-HHL_ glszm_ZoneVariance	gradient_firstorder_Kurtosis	wavelet-HHL_firstorder_ Uniformity
9	wavelet-LHH_ glrlm_RunVariance	wavelet-LHL_glszm_ SizeZoneNonUniformityNormalized	wavelet-HLH_glszm_ LargeAreaHighGrayLevelEmphasis
10	wavelet-LHH_firstorder_Energy	wavelet-HHL_ firstorder_Minimum	wavelet-LHH_ firstorder_Maximum

glcm, gray level cooccurrence matrix; glrlm, gray level run length matrix; glszm, gray level size zone matrix; gldm, gray level dependence matrix.

### Diagnostic Performance of Radiomics

**Figure 3:**
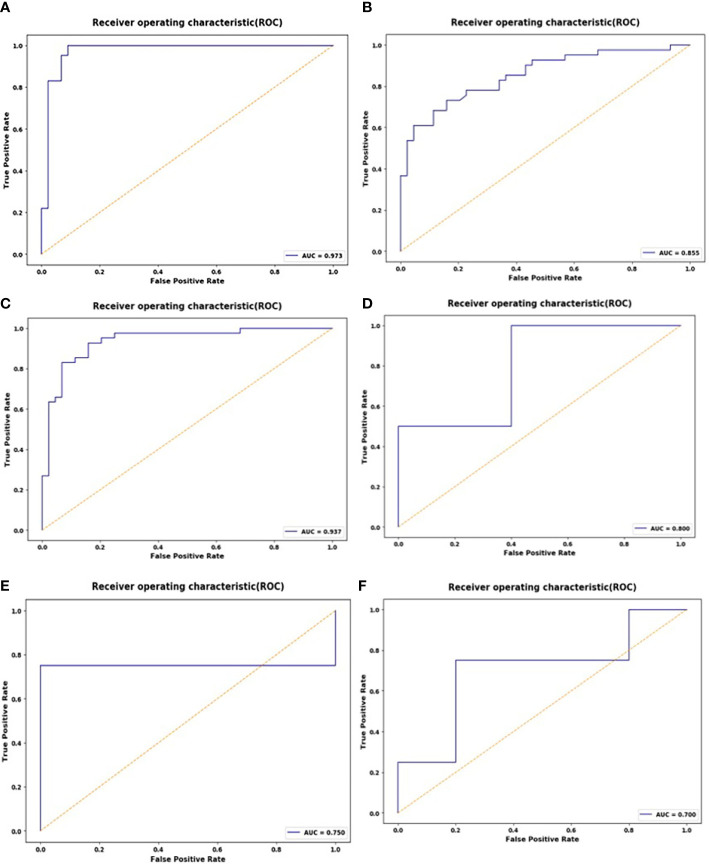
ROC curves of the support vector machine (SVM) classifier in the training set during the non-enhanced phase (NP) **(A)**, arterial phase (AP) **(B)**, and venous phase (VP) **(C)**; receiver operating characteristic (ROC) curves of the SVM classifier in the test set during the NP **(D)**, AP **(E)**, and VP **(F)**.

These 10 selected radiomics features were used to establish the SVM model, which was evaluated by ROC curves. The SVM model achieved good classification performance for differentiating RMS from YSTs. The AUCs of the NP, AP, and VP radiomics models were 0.937 (95% CI: 0.862, 0.978), 0.973 (95% CI: 0.913, 0.996), and 0.855 (95% CI: 0.762, 0.922) in the training set, respectively, which were confirmed in the test set by AUCs of 0.700 (95% CI: 0.328, 0.940), 0.800 (95% CI: 0.422, 0.979), and 0.750 (95% CI: 0.373, 0.962), respectively (**Figure 3**). The accuracy, sensitivity, specificity, and AUC of the radiomics model for each CT phase are shown in **Table 4**.

**Table 4:** Performance of the support vector machine (SVM) classifier for the differential diagnosis of rhabdomyosarcoma (RMS) and yolk sac tumors (YSTs).

Model	AUC (95% Cl)		Accuracy		Sensitivity		Specificity	
	Training set	Test set	Training set	Test set	Training set	Test set	Training set	Test set
NP	0.855	0.700	0.882	0.778	0.927	0.750	0.841	0.800
	(0.762, 0.922)	(0.328, 0.940)						
AP	0.973	0.800	0.953	0.778	1.0	1.0	0.909	0.600
	(0.913, 0.996)	(0.422, 0.979)						
VP	0.855	0.750	0.788	0.889	0.732	0.750	0.841	1.0
	(0.762, 0.922)	(0.373, 0.962)						

## Discussion

Occult tumors located in the basin are difficult to locate on CT images in the presence of large tumors occupying the entire pelvic cavity. RMS comprises a group of highly heterogeneous malignant tumors that can grow anywhere in the body but mainly occur in the retroperitoneal and genitourinary system in the pelvis in children (20). YST is the most common type of germ cell tumor of the pelvis of children. Simple YST is the most common form, mainly originating from the gonads (testes and ovaries), but other types of extragonadal YST can occur in the pelvis, sacral tail, and vagina. (21). The tumor locations of RMS and YSTs partially overlap, and these tumors have similar imaging manifestations, including larger solid tumors occupying the pelvis, with little calcification and hemorrhaging, abundant blood vessels in the AP, and progressive enhancement (21–23). Although some studies have summarized imaging features of RMS, its manifestations still lack specificity (4, 20). Laboratory examination of AFP level is an important indicator for differentiating RMS from YST. In this study, there is significant differences in AFP levels between the two tumors. It is necessary to combine clinical examinations and AFP level to distinguish pelvic germ cell tumors to improve the diagnostic accuracy of RMS.

In recent years, with the development of precision medicine, radiomics has developed rapidly.

Radiomics uses many automatic data characterization algorithms to convert images of areas of interest into quantitative high-throughput feature values. These quantitative features may not be perceivable by the human eye and can reflect the biological information of tumors, such as cell morphology and molecular and gene expression (24). Radiomics provides non-invasive information for diagnosis, differential diagnosis, staging, efficacy evaluation, and prognosis of tumors. Among adult diseases, reports of CT radiomics are more common, including in the differential diagnosis, staging and grading of pancreatic tumors (12), renal cell carcinoma (11), lung cancer (9), and gastric cancer (14). Reports of CT radiomics in children are lacking. In this study, radiomics was combined with machine learning to extract and select quantitative radiomics features derived from NP, AP, and VP CT images of the lesion. Finally, 10 features were selected from each phase as important predictors of radiological characteristics to construct the model. Among the selected features, the texture features were obviously superior to the first-order statistical features, and among the texture features, each phase contained GLSZM features. The GLSZM records the number of occurrences of case where j and i elements are adjacent in the two-dimensional image area and describes the distribution of similar intensity area, which is a measure of the uneven gray level of the tumor area. The GLSZM has a significant value in characterizing texture consistency, aperiodicity, and speckle texture, indicating differences in texture uniformity between RMS and YSTs.

The results showed that radiomics features can distinguish between RMS and YSTs. The classification efficiency of AP CT scans was better than that of the VP and NP scans. The AUC of the AP radiomics model was 0.973 (95% CI: 0.913, 0.996) in the training set, which was confirmed in the test set with an AUC of 0.800 (95% CI: 0.422, 0.979). The reason for this high value may be that the radiomics features extracted from post enhancement AP images can better detect and describe the biological heterogeneity of the tumor. RMS and YSTs are malignant tumors, and the high heterogeneity of malignant tumors may be related to abnormal tumor angiogenesis and cell infiltration. There are abundant blood sinuses and blood vessels between the cell clusters in the AP, and the enhancement is rapid and long-lasting. Therefore, we suggest that radiomics characteristics of the dominant AP in CT scans can be used to distinguish RMS from YSTs, and the VP and delay phase can be omitted in the diagnosis RMS, to reduce the radiation dose.

There were some limitations in this study. First, this was a retrospective study, which may have inherent selection bias. Second, the study did not include other pelvic cell tumors; considering the low incidence of other solid germ cell tumors, RMS was mainly distinguished from YST by clinical radiological diagnosis. Third, the sample size was small. RMS and YST are not common diseases in children; therefore, in future research, we need to further expand the sample size and establish a multicenter, prospective study. Finally, the image layer thickness used in this study was 5 mm, which likely affected the diagnostic performance of radiomics features. We will evaluate the performance differences between thin- and thick-layer images in radiomics analysis in future studies.

## Conclusions

In summary, the CT-based radiomics model developed and validated can be used to effectively distinguish RMS and YST, and combined with clinical features, which can improve diagnostic accuracy and increase the confidence of radiologists in the diagnosis of pelvic solid tumors in children. It is believed that as an important part of precision medicine, radiomics will be widely used in the diagnosis, evaluation, and individualized treatment of diseases in children in the future.

## Data Availability Statement

The raw data supporting the conclusions of this article will be made available by the authors, without undue reservation.

## Ethics Statement

This study was approved by the Ethics Committee of the Children’s Hospital Affiliated to Chongqing Medical University, and the requirement for written informed consent was waived.

## Author Contributions

LH proposed the study. XC and YH performed research, analyzed the data, and wrote the first draft. TZ collected the data, LZ auxiliarily analyzed the data. HD provided all the pathological data and reconfirmed the histologic grade. All authors contributed to the article and approved the submitted version.

## Funding

This study was funded by National Clinical Research Center for Child Health and Disorders (Children's Hospital of Chongqing Medical University, Chongqing, China) (NCRCCHD-2020-EP-04); Basic Research and Frontier Exploration Project (Yuzhong District, Chongqing, China) (NO 20200155).

## Conflict of Interest

The authors declare that the research was conducted in the absence of any commercial or financial relationships that could be construed as a potential conflict of interest.
